# Forecasting Teleconsultation Demand Using an Ensemble CNN Attention-Based BILSTM Model with Additional Variables

**DOI:** 10.3390/healthcare9080992

**Published:** 2021-08-04

**Authors:** Wenjia Chen, Jinlin Li

**Affiliations:** School of Management and Economics, Beijing Institute of Technology, Beijing 100081, China; wenjia-chen@bit.edu.cn

**Keywords:** teleconsultation demand forecasting, ensemble deep learning, convolutional neural networks (CNNs), Baidu Index, air quality index (AQI)

## Abstract

To enhance the forecasting accuracy of daily teleconsultation demand, this study proposes an ensemble hybrid deep learning model. The proposed ensemble CNN attention-based BILSTM model (ECA-BILSTM) combines shallow convolutional neural networks (CNNs), attention mechanisms, and bidirectional long short-term memory (BILSTM). Moreover, additional variables are selected according to the characteristics of teleconsultation demand and added to the inputs of forecasting models. To verify the superiority of ECA-BILSTM and the effectiveness of additional variables, two actual teleconsultation datasets collected in the National Telemedicine Center of China (NTCC) are used as the experimental data. Results showed that ECA-BILSTMs can significantly outperform corresponding benchmark models. And two key additional variables were identified for teleconsultation demand prediction improvement. Overall, the proposed ECA-BILSTM model with effective additional variables is a feasible promising approach in teleconsultation demand forecasting.

## 1. Introduction

By using advanced information and communication technology, teleconsultation sinks high-quality medical resources to underdeveloped areas to alleviate the shortage and uneven distribution of medical resources in China [1,2]. Accurate teleconsultation demand forecasts are important for the efficient utilization of limited teleconsultation resources. To build an accurate forecasting model for daily teleconsultation demand, an ensemble deep learning method was proposed in the previous study [3]. Before the previous study, there wasn’t related literature reporting daily teleconsultation demand forecast. And even relevant operations and management research were few reported. These relevant researches included resource allocation [2], service quality assessment [4], appointment and scheduling [5], workload management [6], and the government subsidy policy [7].

In the previous study of teleconsultation demand forecasting, the proposed forecasting methodology is the ensemble attention-based bidirectional long short-term memory model (EA-BILSTM) [3]. This model was proposed to deal with the sparseness and variability of holiday influence. The variability results from the changed date of Chinese traditional holidays, which is challenging for traditional methods and single algorithms to learn. Empirical studies proved that EA-BILSTM outperformed five traditional methods and four single algorithms. To further enhance forecasting accuracy, several feasible methods can be applied to EA-BILSTM. On the one hand, additional variables are selected to improve the forecast from the perspective of big data, and thus importing more useful information into the forecasting model. As commonly used additional variables, search engine data and weather factors are added to the inputs of forecasting models for many tasks, and the value of additional variables is demonstrated in empirical studies [8,9]. On the other hand, the model structure is improved to enhance the ability of forecasting models and increase forecasting accuracy. For multidimensional data, convolutional neural networks (CNNs) have been proved to be able to extract complex patterns from time-series data, and the learned patterns can significantly improve the forecasting results [9,10,11]. Therefore, CNNs are often added to the recurrent neural network (RNN) to build a more powerful forecasting model.

Given the effectiveness of the two ways mentioned above, this paper attempts to improve the forecasting accuracy of teleconsultation demand by selecting additional variables and adding the CNNs into the built EA-BILSTM model. The main contributions of this paper are described as follows.

(1) This study proposed a novel ensemble hybrid deep learning model to build the daily teleconsultation demand forecast model.
(2) The proposed ensemble hybrid deep learning model, named ECA-BILSTM, combines shallow CNNs, attention mechanisms, and BILSTM. The effectiveness of shallow CNNs on extracting features from multidimensional inputs is proved on actual teleconsultation data.
(3) For the first time, additional variables are selected according to the characteristics of teleconsultation demands and inputted into forecasting models to improve the demand forecasts. The importance of additional variables is identified by comparing model forecasting results.
(4) The ECA-BILSTM with effective additional variables is proved to be a promising tool for teleconsultation demand prediction, which is meaningful for optimizing the use of limited teleconsultation resources.

The rest of the paper is organized as follows. Section 2 gives a review of the related literature. Section 3 describes the experimental data, method and design. Section 4.1, Section 4.2, Section 4.3 and Section 4.4 present the empirical results and discussions, and Section 4.5 draws the main findings. Finally, Section 5 concludes the whole paper and directs future research.

## 2. Literature Review

For several ways of improving time series forecasts, using additional data is an effective way by importing more useful information into the forecasting models. There are many additional data and they can be roughly divided into two categories: the related variables and causal variables. Search engine data is a commonly used related variable for time-series forecasts, especially for the outbreak forecast of epidemics and the demand forecast of tourism. The pioneering work of applying engine query data in time series forecast should be the influenza epidemics detection using Google search queries (Mountain View, CA, USA) [12]. Subsequently, for many other diseases, like dengue [13], norovirus [14], hand-foot-mouth [15], and even the recent COVID-19 [16], search engine data are used to improve the early detection of diseases. In the area of tourism, search engine data are used to improve the forecast of tourist volume. For example, in the forecasts of visitor volumes to Hainan Province, both types of search engine data (Google Trend—Mountain View, CA, USA—and Baidu Index—Beijing, China) helped to significantly decrease forecasting errors compared to the corresponding ARIMA models [8]. To improve tourist attraction management, the tourist flow predictions were improved by using Baidu Index [17,18].

As for the use of causal variables in time series prediction, weather and air pollution data are popularly added to forecasting models of some demands. Weather factors are used in demand forecasting models because they can affect people’s willingness to go out. On bad weather days, people prefer staying at home rather than go out, reducing the demands of some services provided in fixed locations. Therefore, weather factors can be used as effective additional variables for the demand forecast of healthcare services. For example, in short-term ED volume forecasting, the snowfall variable was added to the forecasting model, resulting in improved model performance [19]. In the formulation of predicting the ED patients, the ambient temperature was considered and proved to have differential effects on ED patient visits of different specialties and severities [20]. Also, air pollution factors are often used in healthcare demand forecasting models because air pollution has a direct relationship with people’s healthy states. Severe air pollution will cause respiratory diseases. Therefore, air pollution-related variables are inputted into the demand forecasting model in terms of respiratory diseases [21,22].

As an effective way to improve the time series forecasting accuracy, CNNs are added to forecasting models as feature extractors. CNNs can extract complex spatial or temporal features from input matrices. For example, in the traffic speed prediction, the proposed algorithm applying CNNs has considerable advantages in the predictions compared to other commonly used algorithms [23]. Taking both the self-features and various complex factors of traffic flow into consideration, a CNN-based multi-feature predictive model (MF-CNN) was proposed to obtain accurate traffic flow prediction [9]. Given the RNN models have difficulty in capturing global information and fail to highlight the periodic characteristics of time series, a model with multiple CNNs was proposed to solve the problem of periodic multivariate time-series predictions [10]. In individual-level patient cost prediction, a CNN was used to learn the hidden temporal patterns from multivariate time series data in patient insurance claims [11]. In the task of predicting NO(x) emission during the Fluid Catalytic Cracking process, a deep learning architecture integrating CNN and Long Short-Term Memory Network (LSTM) was built for more accurate prediction [24]. For short-term electricity price prediction, a novel hybrid model, consisting of Variational Mode Decomposition (VMD), CNN, and Gated Recurrent Unit (GRU), was proposed for lower prediction errors. In this hybrid model, CNN was used to further extract the time-domain features for all the intrinsic model functions in the VMD domain [25].

From the above literature review, the use of both additional variables and CNNs can improve time series prediction. Because of the effectiveness of additional variables on prediction improvement for many prediction tasks and no already use of additional variables in teleconsultation demand prediction, we hope to introduce additional variables to improve the prediction accuracy and identify the key additional variables for the improvement of teleconsultation demand prediction. In the studies of applying CNNs in time-series predictions, inputs are often high-dimensional and CNNs are multilayer structures. The structure with multiple layers may not be the proper choice for the model improvement when the inputs are low-dimensional. Therefore, the effectiveness of shallow-layer structured CNNs on extracting features from time series needs to be proved.

## 3. Materials and Methods

### 3.1. Raw Data and Data Preprocessing

The study dataset of this paper is the demand records of teleconsultation collected in the National Telemedicine Center of China (NTCC). The dataset consists of the number of the application from 1 January 2018 to 30 November 2019, with a total of 668 observations. That is, in these 699 days, the records of 31 days were missing.

The missing data is treated by the feature-driven method in the previous study [3]. To make the forecasting results more reliable, two datasets are used in the experiments, namely the 528-day dataset (1 January 2018 to 12 June 2019) and the 699-day dataset (1 January 2018 to 30 December 2019). In the 528-day dataset, the data from 1 January 2018 to 28 February 2019 are used as the training set (accounting for about 80% with 424 observations), and the remaining data are used as the testing set (with 104 observations). In the 699-day dataset, the data from 1 January 2018 to 16 June 2019, are used as the training set (accounting for about 80% with 562 observations), and the remaining data are used as the testing set (with 137 observations).

### 3.2. Additional Variable Selection by the Demand Characteristic

To improve forecasting accuracy, additional variables can be selected and added to forecasting models. But there are so many available additional variables, such as thousands of disease terms for search engine data. To select additional variables efficiently, characteristic of the teleconsultation demand is firstly analyzed here.

In the records of teleconsultation, 65 departments provide the teleconsultation service. Teleconsultation demands of departments are different. The total proportion of the demand of the six departments listed in Table S1 (Supplementary Materials) reaches 50.09%. In those departments, respiratory provides the most services and neurology follows. The services of those two departments are over 4000, much higher than the resting departments.

**Figure 1:**
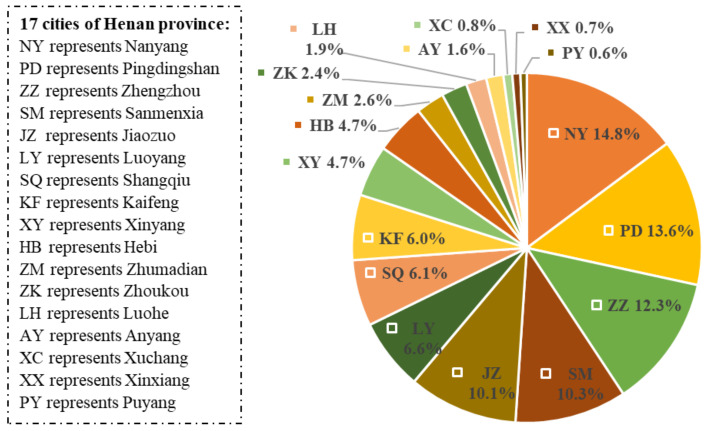
The regional distribution of teleconsultation demand of the respiratory department.

At present, NTCC mainly provides teleconsultation services in Henan province. There are 17 cities in Henan Province, as listed in Figure 1. The teleconsultation demand can come from different regions. Because the respiratory department has the most teleconsultation demand. The regional distribution of the demand of the respiratory department is analyzed and shown in Figure 1. NY has the most 699 demands. The top five cities (NY, PD, ZZ, SM and JZ) in terms of demand volume count 60.2% of the demands. All demand proportions of them are over 10%.

According to the amount characteristic of teleconsultation demand above, additional variables including related variables and causal variables are selected as follows.

#### 3.2.1. Related Variables

In the big data era, massive searching behavior data of netizens can be shared by internet statistical analysis platforms, like Google Trends and Baidu Index. Both of them reflect the popularity of a particular query and users’ interests at a given moment, which can be related to the demand of some services and helpful for demand prediction. Due to the larger market share of Baidu than Google in China, Baidu Index performed better than Google Trends in forecasting tasks regarding China [8]. Baidu Index (http://index.baidu.com/, accessed on 20 August 2020) provides daily Baidu query volume data from June 2006 to the present, and the search data are in absolute numbers. To improve the forecasting accuracy of teleconsultation demand, the Baidu Index of keywords, telemedicine (denoted by “YCYL”) and teleconsultation (denoted by “YCHZ”), are selected as the related variables. Moreover, the Baidu Index of diseases of the top two departments in terms of service volume (respiratory and neurology) are selected as related variables. By simple counting, six Chinese disease terms, as shown in Table S2, are selected as the keywords to collect the Baidu Index data. These Baidu Index data were accessed on 20 August 2020.

#### 3.2.2. Causal Variables

Air pollution affects both physical and mental health [26,27]. The effects of air pollution on healthy are directly reflected by some respiratory symptoms, like coughing. Given the respiratory department has the most demand and five regions (NY, PD, ZZ, SM, and JZ) are the top five regions in terms of the demand volume of the respiratory department, air pollution data of those five regions are used as the causal variables to improve the forecasting results of teleconsultation demand. As a comprehensive air pollution indicator, the air quality index (AQI) observations from 1 January 2018 to 30 November 2019 of the five cities are collected from the National Air Quality Platform (http://106.37.208.233:20035/, accessed on 20 August 2020).

In summary, 13 additional variables are selected to improve the forecasting accuracy of teleconsultation demand, including 8 Baidu Indexes and 5 AQIs. Each of those additional variables will be separately added to the input of forecasting models.

### 3.3. The Proposed Ensemble Hybrid Deep Learning Approach

**Figure 2:**
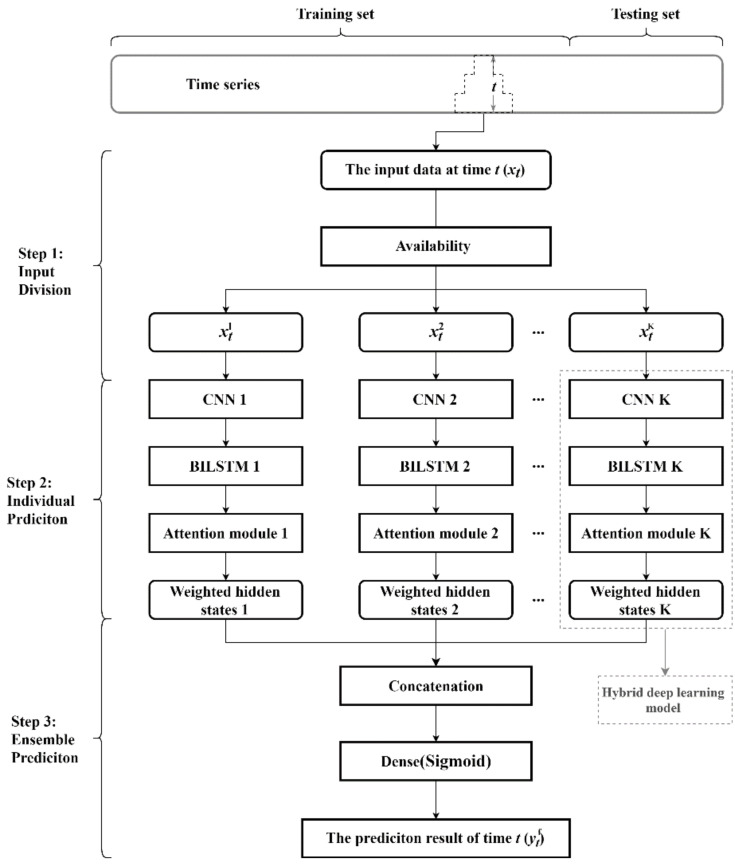
The generic framework of the proposed ECA-BILSTM model.

In this section, a novel ensemble hybrid deep learning approach named ECA-BILSTM is formulated for teleconsultation demand forecasts. The framework of the proposed ECA-BILSTM model is shown in Figure 2. There are three main steps in the methodology.

Step 1: Input Divisions. In the beginning, the input data matrix at time *t* may be in irregular shape because of the different data availability. Data can have different availability at time t because of the different frequencies in data collection or the unknown future information of some variables. According to the different availability, the input data can be divided into different inputting sub-matrices.

Step 2: Individual Prediction. Each input sub-matrix is first inputted into a CNN layer. The CNN is designed to process data that come in the dorm of multiple arrays, like the 1D for signals and sequences, 2D for images [28]. A typical CNN is composed of the input layer, hidden layers and output layer. The multiply hidden layers consist of convolutional layers, activation function layers, pooling layers, and fully connected layers. The convolutional layers, activation function layers and pooling layers map the original data to the feature space of hidden layers. Considering the inputs in teleconsultation demand forecasting are relatively short-lag and low-dimensional, simpler structured CNN, which has less hidden layers with less parameters to be learned, is more suitable for teleconsultation demand forecast. Therefore, the shallow CNN with one convolutional layer and one activation function layer in the hidden layer is applied in the ECA-BILSTM model. Each CNN is a feature extractor to learn the features of multiple variables. The settings of CNNs relate to the size of the input, and the input size depends on the feature of time series, like periodicity. The settings of CNNs in ECA-BILSTM model for teleconsultation forecast are described in Section 3.5. The matrices, learned by CNN from one inputting sub-matrix, are used as the input of a BILSTM with an attention module. Because of the capability of the attention module, the hidden states of BILSTM are weighted and they are seen as the results of the individual prediction.

Step 3: Ensemble Prediction. Attention-based BILSTM outputs weighted hidden states in Step 2. In Step 3, all weighted hidden states are concatenated together as the input of the Dense with Sigmoid activation. Finally, the Dense outputs the demand prediction result of time *t*.

### 3.4. Performance Evaluation Criteria and Statistic Test

To assess the one-step-ahead forecasting accuracy, two criteria are applied, and they are the root mean squared error (RMSE) and the mean absolute error (MAE):

(1) $$RMSE=\sqrt{\frac{1}{N}\overset{N}{\underset{t=1}{\sum }}{\left(x_{t}-{\hat{x}}_{t}\right)}^{2}}\;$$

(2) $$MAE=\frac{1}{N}\overset{N}{\underset{t=1}{\sum }}\left|x_{t}-{\hat{x}}_{t}\right|\;$$

where $x_{t}$ is the actual value, ${\hat{x}}_{t}$ *t* is the predicted value, and *N* is the size of the predictions. To provide statistical evidence of the forecasting ability of the proposed model, the Diebold-Mariano (DM) test was introduced on MAE to identify the significant forecasting differences in models.

### 3.5. The Benchmark Model and Parameter Settings

To test the effectiveness of additional variables and the superiority of the proposed model, the ensemble attention-based BILSTM (EA-BILSTM) model, proposed in the previous study [3], is built and used as the benchmark. The EA-BILSTM has been proved to outperform nine methods, including the traditional econometric model ARIMA, four machine learning models, K Nearest Neighbor (KNN), Support Vector Regression (SVR), Neural Networks (NN), Extreme Learning Machine (ELM), and four deep learning models, Long Short-Term Memory (LSTM), Bi-directional Long Short-Term Memory (BILSTM), Attention-based LSTM (A-LSTM), Attention-based BILSTM (A-BILSTM) [3].

For EA-BILSTM, two inputs are matrix **I_1_** ∈ R ^7 × 8^ and matrix **I_2_** ∈ R ^9 × 7^. In these two inputs, the time lag of history data is 7. The sizes of input metrics are determined by the autocorrelation and partial correlation analyses and the selection of holiday-related variables [3]. For ECA-BILSTM, the parameters of BILSTMs, Dense, and Attention modules are the same as these of EA-BILSTM. The settings of two CNNs in ECA-BILSTM are 7 filters in the CNN treating **I_1_** and 9 filters in the CNN treating **I_2_**, respectively. In two CNNs, the kernel size is 2, the stride is 1, and activation is sigmoid.

## 4. Results

### 4.1. The Effect of Additional Variables on Teleconsultation Demand Forecast

To assess the effect of additional variables, the EA-BILSTM model and the EA-BILSTM model importing additional variables are performed to forecast teleconsultation demand, and the performance comparison results are reported in Table 1 and Table S3. One important conclusion can be obtained that introducing additional variables can improve the forecasting results in teleconsultation demand predictions. Specifically, the lowest RMSE and MAE are achieved on two datasets by importing additional variables into the EA-BILSTM model. However, the DM test results show that the improvements brought by introducing additional variables are not statistically significant. More precisely, the difference between the predictions of EA-BILSTM and EA-BILSTM using one additional variable isn’t statistically significant. Namely, the effectiveness of 13 additional variables is insignificant for the prediction improvement of EA-BILSTM on teleconsultation demand.

Besides, other exiting methods, including KNN, SVR, NN, LSTM and BILSTM, are performed to forecast teleconsultation demand, and the forecasting results are presented in Table S4. From the MAE in Table S4, most of the selected additional variables can’t make KNN, SVR and NN obtain better forecasting performance, while can make LSTM and BILSTM obtain better performance. But in terms of teleconsultation demand forecast, EA-BILSTM can outperform KNN, SVR, NN, LSTM and BILSTM no matter whether the additional variables are used. Therefore, the EA-BILSTM is used as the benchmark model in the forecasting performance comparison of following sections.

### 4.2. The Superiority of the ECA-BILSTM

Importing additional variables can’t make EA-BILSTM achieve statistically better performance on the teleconsultation demand forecast. A possible explanation for this failure is that the employed algorithms can’t handle more complex data. To overcome this defect, the ECA-BILSTM is built, in which shallow-structured CNN layers are used as filters and extract useful information from complex data to improve the demand forecast.

**Figure 3:**
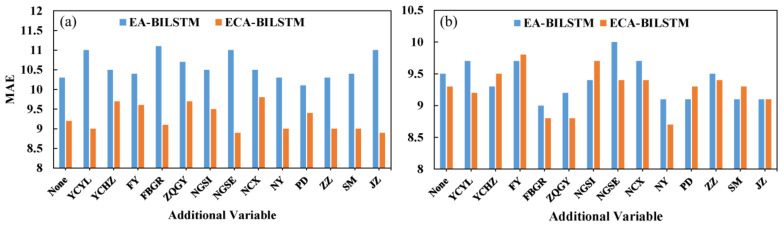
The MAE of EA-BILSTM and ECA-BILSTM on (**a**) 528-day dataset and (**b**) 699-day dataset.

For the prediction accuracy of ECA-BILSTM with additional variables, Table 2 reports the results of the RMSE and MAE criteria on two datasets. Two main important conclusions can be obtained from Table 2. First, ECA-BILSTM can achieve better forecasting accuracy than EA-BILSTM no matter whether the additional variables are used. The MAE of EA-BILSTM and ECA-BILSTM are compared in Figure 3. Without additional variables, ECA-BILSTM has lower MAE than that of EA-BILSTM on both two datasets. With additional variables, on the one hand, ECA-BILSTM consistently outperforms EA-BILSTM on the 528-day dataset. These results illustrate the superiority of ECA-BILSTM. The main hidden reason could be that the CNN layers enhance the ability of the built model to deal with more complex inputs. These results indicate that the proposed ECA-BILSTM model can be used as a promising tool for the teleconsultation demand forecast. On the other hand, not all additional variables can make ECA-BILSTM outperform EA-BILSTM. For example, some MAE of ECA-BILSTM in the right subfigure of Figure 3 are higher than the corresponding MAE of EA-BILSTM. The effect of additional variables on ECA-BILSTM will be analyzed in Section 4.3.

Second, using additional variables can improve the forecasting accuracy of ECA-BILSTM. Lower RMSE and MAE criteria on two datasets are obtained by importing additional variables into the ECA-BILSTM. In particular, the AQI data of NY and JZ respectively make ECA-BILSTM achieve the best performance on the two datasets. And those forecasting results are also the best-performed among all results from used models, showing improvement on forecasting brought by the imported AQI additional variables and added CNN structure in the forecasting model. In addition, it can be inferred that the AOI data perform better than Baidu Index data on teleconsultation demand forecasting. This can be explained that the poor air quality presented by AQI is directly harmful to respiratory health, which can increase the demands of healthcare services of the respiratory department. The respiratory department has the most demand for teleconsultation. Therefore, the AOI data can effectively improve teleconsultation demand prediction.

To further illustrate the superiority of ECA-BILSTM, the DM test is applied to MAE. The DM test results of MAE between EA-BILSTM models and corresponding ECA-BILSTM models are shown in Table S5. The bolded DM test results in Table S5 represent the statistical outperformance of ECA-BILSTM models compared to the corresponding EA-BILSTM models. When using additional variables YCYL and ZQGY, the improvement of forecasting accuracy brought by ECA-BILSTM on two datasets is significant. This result further proves the advancement of the architecture of ECA-BILSTM than that of EA-BILSTM.

### 4.3. The Forecasting Improvement of ECA-BILSTM Model with Additional Variables

From Section 4.1 and Section 4.2, it is known that the effectiveness of additional variables on improving the forecasting performance of EA-BILSTM is non-significant, but ECA-BILSTMs can statistically outperform EA-BILSTMs. This section would like to analyze the forecasting improvement of ECA-BILSTM with additional variables. For this purpose, the DM test is applied between EA-BILSTM without additional variables and ECA-BILSTM with additional variables. The DM test results are shown in Table 3, and three main conclusions can be found.

First, ECA-BILSTM with additional variables can obtain significantly better predictions than EA-BILSTM. Both of the imported additional variables and added CNN structure into the model play key roles in the forecasting improvement. For example, the prediction difference between EA-BILSTM and EA-BILSTM-NY isn’t significant; the prediction difference between EA-BILSTM-NY and ECA-BILSTM-NY isn’t significant, but the prediction difference between EA-BILSTM and ECA-BILSTM-NY is significant. This result demonstrates the synergy of the imported additional variable and added CNN structure in the model. A likely explanation is the additional variables can add extra useful information into models and meanwhile CNN layers can strengthen the ability of the model to treat complex data for the improvement of teleconsultation demand prediction.

Second, additional variables should be carefully used to improve the teleconsultation demand forecast. Although most of the forecasting results of ECA-BILSTM using additional variables are better than that of EA-BILSTM, some of those promotions are non-significant. The effect of different additional variables on ECA-BILSTM prediction performance is different. How to select the suitable ones needs further research.

Third, important additional variables for teleconsultation demand prediction can be identified. The MAE criteria of ECA-BILSTM on both two datasets are statistically better by using the Baidu Index of ZQGY and the AQI data of NY. Therefore, the Baidu Index of ZQGY and the AQI data of NY can be identified as effective and key additional variables to improve teleconsultation demand prediction. By using key additional variables, the forecasting accuracy of ECA-BILSTM can be significantly improved. The proposed ECA-BILSTM with effective additional variables can be used as a quite promising tool for teleconsultation demand forecast.

### 4.4. Discussions

From the above experiment results, the superiority of the ECA-BILSTM model is proved. And the effectiveness of different additional variables on teleconsultation demand prediction improvement is different. Among 13 additional variables, the Baidu Index of ZQGY and the AQI data of NY are identified as the key additional variables to improve teleconsultation demand prediction. In this section, the superiority of the ECA-BILSTM model and the effectiveness of the Baidu Index of ZQGY and the AQI of NY are discussed below.

As for adding CNNs layers into the forecasting models, several studies aimed at using them as the extractors for a variable number of patterns with various shapes in time series. And the CNN blocks have been empirically proved to be able to extract complex information. Due to this capacity, the teleconsultation demand forecasting accuracy can be significantly improved by adding CNNs into the EA-BILSTM model. As so far, CNNs has successfully improved predictions by extracting specific patterns in time series, such as temporal and spatial characteristics of traffic flows [9,23,29,30], periodic characteristics of solar-energy [10], hidden temporal patterns of patient insurance claims [11], time-domain features of the intrinsic model functions decomposed from electricity prices [25], static and dynamic characteristics of gold [31]. Moreover, the model inputs of teleconsultation forecast are low-dimensional. Thus, the shallow CNNs are enough powerful to enhance the demand forecast.

**Figure 4:**
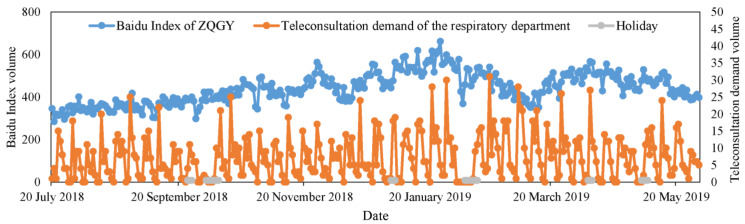
The changes of the Baidu Index of ZQGY and teleconsultation demand of respiratory department with the date.

Concerning the effectiveness of the two additional variables (the Baidu Index of ZQGY and the AQI data of NY) for the teleconsultation demand forecast, there are two reasonable explanations. On the one hand, the Baidu Index of ZQGY as one related variable, to some extent, has a similar variety with the teleconsultation demand. Figure 4 shows the changes of the Baidu Index of ZQGY and the teleconsultation demand of the respiratory department with the date. The ZQGY represents bronchitis, one of the common diseases in the respiratory department. From Figure 4, it can be observed that there is a long-term relationship between the Baidu Index of ZQGY and the teleconsultation demand of the respiratory department. For example, when the Baidu query volume of ZQGY increases from January 2019 to March 2019, the peaks of respiratory demands show delayed increases. Such complex long-term relationships are challenging for models to learn. But the proposed ECA-BILSTM exhibits the ability to handle the complexity between input variables.

On the other hand, the AQI data as one causal variable can help forecast the demand of healthcare service of the respiratory department in the following days because the poor air quality displayed by high values of AQI can directly damage respiratory health. Besides, the regional distribution of the teleconsultation demand of respiratory shows that the NY region has the most demand. Therefore, the AQI data of NY presents significant effectiveness in improving the teleconsultation demand forecasts of ECA-BILSTM.

To further prove the effectiveness of the Baidu Index of ZQGY and the AQI data of NY on teleconsultation demand forecast, the prediction accuracy of EA-BILSTM and ECA-BILSTM using them on respiratory department demand is measured and the results are shown in Table S6. From Table S6, it can be observed that adding the Baidu Index of ZQGY and the AQI data of NY into EA-BILSTM can obtain better forecasting results. And ECA-BILSTMs can achieve better forecasting performance than EA-BILSTMs. The DM test results of MAE of respiratory department demand prediction are presented in Table S7. The superiority of the ECA-BILSTM model and the forecasting improvement of the ECA-BILSTM model with additional variables are further proved. The Baidu Index of ZQGY and the AQI data of NY show their effectiveness on respiratory department demand prediction.

### 4.5. Summarization

From the above experimental results and result discussions, four main conclusions can be drawn as follows. (1) The proposed ECA-BILSTM can be tested significantly superior to the benchmark EA-BILSTM in terms of prediction accuracy. (2) When using different additional variables, the ECA-BILSTM models can significantly outperform their corresponding EA-BILSTM models, indicating the effectiveness of the CNN layers in model improvement to deal with complex data. In particular, shallow CNN layers are proved to be able to extract features from low-dimensional inputs. (3) Two key additional variables, the Baidu Index of ZQGY and the AQI data of NY, are identified for the improvement of the teleconsultation demand forecasts. (4) The proposed ECA-BILSTM model with effective additional variables is a promising approach in teleconsultation demand forecasting.

## 5. Conclusions

Due to the availability of more and more data, additional variables including related variables and causal variables can be used in teleconsultation demand forecasting. To enhance the ability to deal with the higher complexity of inputs resulting from using additional variables, an ensemble hybrid deep learning model named ECA-BILSTM is proposed in this study. In this approach, shallow CNN layers are added to the EA-BILSTM to extract the multi-features among multidimensional data. In empirical studies, the ECA-BILSTM models can outperform the corresponding EA-BILSTM models. Two additional variables, the Baidu Index of ZQGY and the AQI data of NY, are identified as the effective and key additional variables to improve the teleconsultation demand forecast. DM test results further confirm the superiority of the proposed model and the effectiveness of the key additional variables. The proposed approach ECA-BILSTM with proper additional variables can be used as a promising forecasting tool for teleconsultation demand.

Despite the capability of the ECA-BILSTM model and the improvement of importing the effective additional variables into the model, there is still room for improvement. For example, in the additional variables used in this study, the disease keywords of the Baidu Index are selected by simply counting the number of occurrences in the initial diagnosis text. This is not the best way to choose the keywords to collect Baidu Index data, which can be optimized by text mining of the initial diagnosis.

## Figures and Tables

**Table 1:** Prediction accuracy of EA-BILSTM.

Additional Variable		Model	528-Day Dataset		699-Day Dataset	
			RMSE	MAE	RMSE	MAE
None		EA-BILSTM	14.7	10.3	12.9	9.5
Baidu Index	YCYL	EA-BILSTM-YCYL	15.7	11.0	13.7	9.7
	YCHZ	EA-BILSTM-YCHZ	15.0	10.5	13.1	9.3
	FY	EA-BILSTM-FY	15.1	10.4	13.0	9.7
	FBGR	EA-BILSTM-FBGR	15.4	11.1	**12.4**	**9.0**
	ZQGY	EA-BILSTM-ZQGY	14.8	10.7	**12.7**	**9.2**
	NGSI	EA-BILSTM-NGSI	14.8	10.5	13.1	**9.4**
	NGSE	EA-BILSTM-NGSE	16.3	11.0	13.6	10.0
	NCX	EA-BILSTM-NCX	15.6	10.5	13.1	9.7
AQI	NY	EA-BILSTM-NY	15.3	10.3	**12.9**	**9.1**
	PD	EA-BILSTM-PD	14.7	**10.1**	**12.3**	**9.1**
	ZZ	EA-BILSTM-ZZ	**14.3**	**10.3**	13.0	9.5
	SM	EA-BILSTM-SM	15.6	10.4	**12.6**	**9.1**
	JZ	EA-BILSTM-JZ	15.5	11.0	**12.8**	**9.1**

Notes: The bold results are lower than the results of EA-BILSTM. The underlines are the best results in each column.

**Table 2:** Prediction accuracy of ECA-BILSTM.

Additional Variable		Model	528-Day Dataset		699-Day Dataset	
			RMSE	MAE	RMSE	MAE
None		ECA-BILSTM	12.7	9.2	12.5	9.3
Baidu Index	YCYL	ECA-BILSTM-YCYL	**12.4**	**9.0**	12.6	**9.2**
	YCHZ	ECA-BILSTM-YCHZ	13.5	9.7	12.9	9.5
	FY	ECA-BILSTM-FY	13.7	9.6	13.0	9.8
	FBGR	ECA-BILSTM-FBGR	13.1	9.1	**12.2**	**8.8**
	ZQGY	ECA-BILSTM-ZQGY	13.5	9.7	**12.2**	**8.8**
	NGSI	ECA-BILSTM-NGSI	13.1	9.5	13.3	9.7
	NGSE	ECA-BILSTM-NGSE	**12.5**	**8.9**	12.6	9.4
	NCX	ECA-BILSTM-NCX	13.5	9.8	12.9	9.4
AQI	NY	ECA-BILSTM-NY	**12.5**	**9.0**	**12.2**	**8.7**
	PD	ECA-BILSTM-PD	13.0	9.4	12.8	**9.3**
	ZZ	ECA-BILSTM-ZZ	**12.7**	**9.0**	12.9	9.4
	SM	ECA-BILSTM-SM	13.3	9.0	12.8	9.3
	JZ	ECA-BILSTM-JZ	**12.1**	**8.9**	12.6	**9.1**

Notes: The bold results are lower than the results of ECA-BILSTM. The underlines are the best results in each column.

**Table 3:** The DM test results of MAE between EA-BILSTM and ECA-BILSTM.

Benchmark Model	Test Model	DM Test Results	
		528-Day Dataset	699-Day Dataset
EA-BILSTM	ECA-BILSTM-YCYL	**1.69**	1.36
EA-BILSTM	ECA-BILSTM-YCHZ	0.85	−0.09
EA-BILSTM	ECA-BILSTM-FY	0.94	−1.06
EA-BILSTM	ECA-BILSTM-FBGR	**2.79**	1.52
EA-BILSTM	ECA-BILSTM-ZQGY	**2.25**	**1.88**
EA-BILSTM	ECA-BILSTM-NGSI	1.11	0.32
EA-BILSTM	ECA-BILSTM-NGSE	1.59	0.60
EA-BILSTM	ECA-BILSTM-NCX	1.05	0.52
EA-BILSTM	ECA-BILSTM-NY	**1.93**	**2.10**
EA-BILSTM	ECA-BILSTM-PDS	0.85	−0.91
EA-BILSTM	ECA-BILSTM-ZZ	**2.09**	0.15
EA-BILSTM	ECA-BILSTM-SMX	**1.86**	0.52
EA-BILSTM	ECA-BILSTM-JZ	1.51	1.13

Notes: In the test statistics, the bold results are statistically significant.

## Data Availability

The data underlying this article were provided by the National Telemedicine Center of China under license. Data will be shared on request to the corresponding author with permission of the National Telemedicine Center of China.

## Supplementary Materials

The following are available online at https://www.mdpi.com/article/10.3390/healthcare9080992/s1, Table S1: Six departments providing the majority of teleconsultation services; Table S2: Selected disease keywords for Baidu Index data; Table S3: The DM test results of MAE between the EA-BILSTM and the EA-BILSTM using one additional variable; Table S4: MAE of existing methods for teleconsultation demand prediction on the 528-day dataset; Table S5: The DM test results of MAE between EA-BILSTM models and the corresponding ECA-BILSTM models; Table S6: Prediction accuracy of EA-BILSTM and ECA-BILSTM on teleconsultation demand of the respiratory department; Table S7: The DM test results of MAE on the respiratory department demand prediction.
