# Supplementary material for: Forecasting Teleconsultation Demand Using an Ensemble CNN Attention-Based BILSTM Model with Additional Variables
Source: Healthcare (Basel). 2021 Aug 4;9(8):992. doi: 10.3390/healthcare9080992 (PMC8391747; doi:10.3390/healthcare9080992)
Supplement: Supplementary file 1 [file healthcare-09-00992-s001.zip › healthcare-1291840-supplementary.pdf]

healthcare

MDPI

Forecasting Teleconsultation Demand Using an Ensemble CNN Attention-based BILSTM Model with Additional Variables

Table S1. Six departments providing the majority of teleconsultation services.

Department	Service (times) ↓	Percentage (%) ↓
Respiratory	4800	13.63
Neurology	4035	11.46
Pediatrics	2791	7.93
Oncology	2264	6.43
Orthopedics	2047	5.81
Cardiology	1699	4.83

Table S2. Selected disease keywords for Baidu Index data.

Department	Disease Term	Notation in This Paper	The Number of Occurrences in The Initial Diagnosis Text↓
Respiratory	Pneumonia	FY	2063
	Lung infection	FBGR	1769
	Bronchitis	ZQGY	558
Neurology	Cerebral infarction	NGSI	1251
		NGSE	846
	Cerebral hemorrhage	NCX	131

Table S3. The DM test results of MAE between the EA-BILSTM and the EA-BILSTM using one additional variable.

Benchmark model	Test Models	DM test results	
		528-Day Dataset	699-Day Dataset
EA-BILSTM	EA-BILSTM-YCYL	−0.84	−1.01
EA-BILSTM	EA-BILSTM-YCHZ	−0.23	0.45
EA-BILSTM	EA-BILSTM-FY	−0.13	−0.49
EA-BILSTM	EA-BILSTM-FBGR	−1.52	1.47
EA-BILSTM	EA-BILSTM-ZQGY	−0.54	1.30
EA-BILSTM	EA-BILSTM-NGSI	−0.25	0.13
EA-BILSTM	EA-BILSTM-NGSE	−0.70	−1.39
EA-BILSTM	EA-BILSTM-NCX	−0.26	−0.57
EA-BILSTM	EA-BILSTM-NY	−0.02	0.99
EA-BILSTM	EA-BILSTM-PD	0.82	1.04
EA-BILSTM	EA-BILSTM-ZZ	0.11	−0.10
EA-BILSTM	EA-BILSTM-SM	−0.16	0.50
EA-BILSTM	EA-BILSTM-JZ	−1.52	0.79

Table S4. MAE of existing methods for teleconsultation demand prediction on the 528-day dataset.

Additional Variable		Model					
		ARIMA	KNN	SVR	NN	LSTM	BILSTM
None		14.03	13.3	13.8	13.1	12.8	11.1
Baidu Index	YCYL		13.9	15.1	14.3	12.1	11.6
	YCHZ		13.8	14.6	15.1	11.7	11.0
	FY		14.0	14.2	15.0	12.9	11.0
	FBGR		13.9	14.4	14.6	13.6	10.8
	ZQGY		<u>13.0</u>	<u>13.5</u>	14.6	11.6	10.7
	NGSI		13.2	13.9	15.5	12.6	13.1
	NGSE		13.2	13.9	14.1	12.9	12.6
	NCX		13.4	13.9	14.0	14.2	11.6
AQI	NY		13.5	14.9	<u>12.9</u>	<u>11.0</u>	11.3
	PD		13.3	14.1	13.8	11.4	11.0
	ZZ		13.4	14.1	13.5	11.8	<u>10.6</u>
	SM		13.4	13.8	13.5	12.6	10.9
	JZ		13.2	14.0	13.5	11.5	12.1

Note: The underlines are the best results in each column.

Table S5. The DM test results of MAE between EA-BILSTM models and the corresponding ECA-BILSTM models.

Benchmark Model	Test Models	DM Test Results	
		528-Day Dataset	699-Day Dataset
EABILSTM	ECA-BILSTM	**1.71**	0.43
EA-BILSTM-YCYL	ECA-BILSTM-YCYL	**2.02**	**1.66**
EA-BILSTM-YCHZ	ECA-BILSTM-YCHZ	0.78	−0.47
EA-BILSTM-FY	ECA-BILSTM-FY	1.02	−0.14
EA-BILSTM-FBGR	ECA-BILSTM-FBGR	**4.89**	0.44
EA-BILSTM-ZQGY	ECA-BILSTM-ZQGY	**3.18**	**1.68**
EA-BILSTM-NGSI	ECA-BILSTM-NGSI	1.18	0.12
EA-BILSTM-NGSE	ECA-BILSTM-NGSE	**1.84**	1.27
EA-BILSTM-NCX	ECA-BILSTM-NCX	0.87	1.06
EA-BILSTM-NY	ECA-BILSTM-NY	1.52	0.71
EA-BILSTM-PD	ECA-BILSTM-PD	0.63	−1.05
EA-BILSTM-ZZ	ECA-BILSTM-ZZ	**2.43**	0.17
EA-BILSTM-SM	ECA-BILSTM-SM	1.56	−0.38
EA-BILSTM-JZ	ECA-BILSTM-JZ	**2.63**	0.10

Note: In the test statistics, the bold results are statistically significant.

Table S6. Prediction accuracy of EA-BILSTM and ECA-BILSTM on teleconsultation demand of the respiratory department.

Models	528-Day Dataset		699-Day Dataset	
	RMSE	MAE	RMSE	MAE
EA-BILSTM	4.34	3.07	3.55	2.52
EA-BILSTM-ZQGY	**4.33**	**3.02**	**3.24**	**2.32**
EA-BILSTM-NY	4.57	**3.02**	**<u>2.94</u>**	**<u>2.13</u>**
ECA-BILSTM	**<u>4.05</u>**	**2.91**	**3.03**	**2.28**
ECA-EABILSTM-ZQGY	**<u>4.05</u>**	**<u>2.88</u>**	**2.97**	**2.22**
ECA-EABILSTM-NY	**4.26**	**2.95**	**3.05**	**2.27**

Notes: The bold results are lower than the results of EA-BILSTM. The underlines are the best results in each column.

Table S7. The DM test results of MAE on the respiratory department demand prediction.

Benchmark models	Test Models	DM Test Result	
		528-Day Dataset	699-Day Dataset
EA-BILSTM	EA-BILSTM-ZQGY	0.17	1.12
EA-BILSTM	EA-BILSTM-NY	0.30	1.43
EA-BILSTM	ECA-BILSTM	**1.69**	1.56
EA-BILSTM-ZQGY	ECA-BILSTM-ZQGY	0.47	0.96
EA-BILSTM-NY	ECA-BILSTM-NY	0.55	−0.97
EA-BILSTM	ECA-BILSTM-ZQGY	1.54	**1.78**
EA-BILSTM	ECA-BILSTM-NY	0.66	**1.72**

Note: In the test statistics, the bold results are statistically significant.
